# The potential distributional health and financial benefits of increased tobacco taxes in Ethiopia: Findings from a modeling study

**DOI:** 10.1016/j.ssmph.2022.101097

**Published:** 2022-04-14

**Authors:** Averi Chakrabarti, Solomon Tessema Memirie, Seblewongel Yigletu, Mizan Kiros Mirutse, Stéphane Verguet

**Affiliations:** a Department of Global Health and Population, Harvard T.H. Chan School of Public Health, 677 Huntington Avenue, Boston, MA, 02115, USA; b Department of Pediatrics and Child Health, College of Health Sciences, Addis Ababa University, NBH1, 4killo King George VI Street, Addis Ababa, Ethiopia; c Federal Ministry of Health of Ethiopia, 1234 Sudan Street, Addis Ababa, Ethiopia

**Keywords:** Tobacco control, Cigarettes, Taxes, Ethiopia, Sub-Saharan Africa, Equity, Distributional impact

## Abstract

Ethiopia raised taxes on tobacco products in early 2020, increasing the overall price of the typical pack of cigarettes by about 67%. We quantify the potential impacts of Ethiopia's tobacco tax hike on various outcomes—life years, tax revenues, cigarette expenditures and catastrophic health expenditures (CHE). Using parameters like price elasticity of demand for cigarettes and smoking prevalence in Ethiopia from the existing literature and secondary data sources, we model the potential implications of the reform at the population level and for different wealth quintiles. We focus only on men since a small proportion of Ethiopian women smoke. Results indicate that Ethiopia's tax hike could induce a significant proportion of current smokers to quit smoking and thereby save almost eight million years of life in the current population. The reform is also likely to increase tax revenues by USD26 million in the first year after its introduction. The richest quintile will bear the greatest share of this higher tax burden and the poorest will bear the least. Additionally, deaths due to the main diseases associated with smoking will fall. This is expected to avert up to 173,000 CHE cases due to the out-of-pocket costs that would have been incurred in obtaining medical treatment. This analysis highlights that cigarette tax hikes in countries that have low smoking prevalence can reduce smoking even further, and thereby protect against the future health and financial costs of smoking. Importantly, the effects of these policies can be progressive across the income spectrum.

## Highlights

• We model the potential impacts of a recent tobacco tax increase in Ethiopia.
• Results show that the reform could save eight million years of life.
• Government tax revenue will likely go up by $26 million in the first year.
• Up to 173,000 fewer cases of catastrophic health expenditures could occur.
• All these effects are expected to disproportionately benefit the poorest.

## 1 Introduction

Tobacco use is relatively low in Ethiopia, the second most populated country in Africa (United Nations Population Division, 2019). Among men (aged 15 years or more), 9% use tobacco products and among women, only 1% use tobacco (WHO, 2021b). Given low usage, tobacco is not currently one of the main drivers of total mortality and morbidity in Ethiopia (IHME, 2019) but it does impose significant costs on society, with healthcare expenditures and lost productivity possibly amounting to 1,400 million Ethiopian Birr (ETB) per year (The Tobacco Atlas, 2018). If left unregulated, poor health outcomes due to smoking could grow to be a bigger public health challenge. A factor that increases the possibility of such a contingency is Ethiopia's large youth population—children under the age of 15 years account for 40% of the population (UN Population Fund, 2021). As smoking becomes more socially acceptable, a large proportion of young cohorts could initiate smoking, with consequent health issues likely to emerge several decades later. Another cause for concern is the concerted effort being made by global tobacco companies to expand into African markets: population growth and the relatively lax tobacco control environment in these settings provide opportunities for boosting tobacco sales (Baleta, 2010; Guliani et al., 2019). In fact, as of 2016, Japan Tobacco International (JTI) has acquired a significant portion of the shares of the leading national tobacco company in Ethiopia (Erku & Tesfaye, 2019). Since tobacco companies often use aggressive marketing strategies and try to shape the tobacco policy environment to their advantage, the entry of a powerful global actor into Ethiopia's tobacco industry could pave the way to higher levels of tobacco consumption (Guliani et al., 2019).

The World Health Organization (WHO) has developed a policy package to provide guidance to countries on the cost-effective measures that can be used to reduce the dangers of tobacco. Known as MPOWER, this set of strategies recommends: monitoring tobacco usage; protecting populations from tobacco smoke inhalation; offering assistance with smoking cessation; warning about tobacco harms; enforcing bans on tobacco advertisements and promotions; and raising tobacco taxes (WHO, 2021a). Ethiopia has recently started implementing such tobacco control strategies (see webappendix Table A1 for a timeline of tobacco-related legislation in the country). In 2014, the country ratified WHO's Framework Convention on Tobacco Control (FCTC) (Erku & Tesfaye, 2019). Subsequently in 2019, the country introduced comprehensive tobacco control legislation mandating smoke-free public spaces, bans on tobacco advertisements, restrictions on the sale of tobacco products and the inclusion of warning labels on tobacco products. The legislation also prohibited the sale of tobacco to youth below the age of 21 years (Federal Democratic Republic of Ethiopia, 2019b; Marquez, 2019). While Ethiopia has clearly taken steps to meet several of the MPOWER policy recommendations and strategies, it has not made much progress on providing smoking cessation services and treatments for tobacco addiction. To illustrate, the availability of nicotine replacement therapies is quite limited in Ethiopia (Erku et al., 2019; Erku & Tesfaye, 2019).

**Fig. 1:**
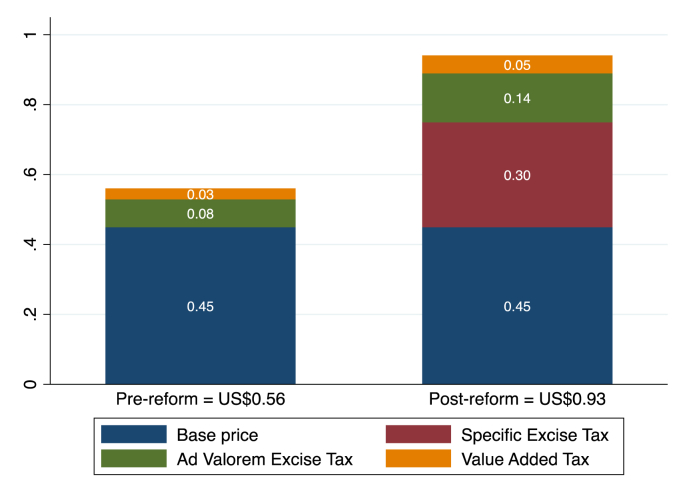
Pre- and post-reform price components of a cigarette pack in Ethiopia. Note: Prices are reported in 2019 USD. Source: WHO (2019).

The current study focuses on the tobacco reform that was passed in Ethiopia in early 2020 to increase taxes imposed on tobacco products (Stoklosa, 2020). Prior to this tax hike, the price of a pack of the most commonly sold cigarette brand was about $0.56 (in 2019 USD). As is depicted in Fig. 1, taxes accounted for about 19% of the total price—ad valorem excise taxes were 14% and value-added taxes were 5% (WHO, 2019). The 2020 tax reform stipulated that excise taxes would now constitute 30% of the production costs of domestic cigarettes or 30% of the cost, insurance and freight (CIF) of imported cigarettes. An additional ETB8 (or USD0.30) was imposed on every pack of cigarettes (Stoklosa, 2020). If the base price (total price minus tax) of the most common brand stays constant, the price per pack would increase to about USD0.93, of which total taxes would be USD0.48 or about 51%. We estimate that the overall price per pack would increase by 67%.

In this analysis, we study the manifold potential effects of the 2020 increase in Ethiopia's tobacco tax. Specifically, we estimate the likely impacts on four different outcomes: years of life gained (YLG) for those induced to cease smoking and for those prevented from initiating smoking; increase in tax revenues; net change in cigarette expenditures for consumers; and financial risk protection via catastrophic health expenditures (CHE) averted due to the reduction in out-of-pocket (OOP) treatment costs for tobacco-related diseases. Tobacco tax increases are often criticized as being regressive (Verguet et al., 2021) and accordingly, we probe results along the wealth spectrum so as to understand whether Ethiopia's reform would indeed impose disproportionate harms on the poorest sections of society.

## 2 Methods

### 2.1 Data inputs

Data and input parameters were extracted from the Ethiopian Demographic and Health Survey (DHS) and the published literature. We used the 2016 round of the Ethiopian DHS (Central Statistical Agency (CSA) Ethiopia and ICF, 2016). Note that this pertains to the period shortly before the tobacco tax hike and therefore its data are used to approximate baseline values on the variables of interest. Covering nationally representative samples, the DHS provides information on a wide range of population, health and nutrition indicators. Data are collected with household questionnaires, and individual questionnaires administered to women aged 15–49 years and to men aged 15–59 years (Central Statistical Agency (CSA) Ethiopia and ICF, 2016).

Less than 1% of women smoke in Ethiopia (Central Statistical Agency (CSA) Ethiopia and ICF, 2016) and so we focused on men in our analysis. Based on the composition of households surveyed by the DHS, we estimated the distribution of the country's male population across different age ranges. Since the DHS categorizes all surveyed households into wealth quintiles, we were further able to approximate the proportion of the country's total population constituted by men in each age-wealth grouping (Table 1, Panel A).

**Table 1:** Input parameters used in the impact model by age and wealth.

*Panel A: Male population distribution (proportions)*					
	Poorest	Poorer	Middle	Richer	Richest
<15	0.054	0.052	0.049	0.046	0.035
15–24	0.011	0.013	0.014	0.017	0.018
25–34	0.010	0.011	0.011	0.011	0.017
35–44	0.007	0.008	0.009	0.009	0.010
45–59	0.006	0.006	0.007	0.007	0.007
*Panel B: Male smoking prevalence (proportions)*					
	Poorest	Poorer	Middle	Richer	Richest
15–24	0.038	0.052	0.018	0.016	0.032
25–34	0.187	0.116	0.115	0.047	0.081
35–44	0.205	0.212	0.155	0.066	0.171
45–59	0.156	0.102	0.168	0.101	0.144
*Panel C: Averageper capitadaily cigarette consumption*					
	Poorest	Poorer	Middle	Richer	Richest
15–24	4.29	4.85	2.38	2.19	5.57
25–34	3.38	5.54	2.35	2.98	9.24
35–44	4.14	5.11	3.33	3.40	5.10
45–59	6.64	3.98	8.81	4.84	9.80

Source: The 2016 Ethiopian Demographic and Health Survey (DHS). Sample weights used for all statistics derived from the DHS.

Information on smoking prevalence and cigarette consumption levels came from the DHS individual male questionnaire. Table 1 (Panel B) presents the approximate proportion of men who smoke across age and wealth categories. To obtain the total number of smokers in each group at baseline, we combined information on population proportions and smoking prevalence from the DHS with total country population in 2016 (around 100 million) (World Bank, 2020). We present average per capita daily cigarette consumption in Table 1, Panel C. Note that we present statistics for broad age groups (Table 1; webappendix Table A2 contains parameters for the finer age groupings that we examined).

The DHS individual questionnaires are administered to respondents aged 15 years and above and so we did not have data on tobacco use for younger individuals. We assumed that children and youth below 15 years are yet to initiate smoking but that in the absence of the tax increase, their future tobacco use patterns would have been akin to those observed amongst individuals currently aged 15–24 years (a conservative assumption, as in Verguet et al., 2015) (specifically, youth are assigned the average of each tobacco use measure across the 15–19 and 20–24 year age groups). Further, we assumed that non-smokers aged 15 years and older would not have initiated smoking in the future. We restricted our examination to men up to the age of 59 years as this is the DHS’ upper age limit for the interview of male respondents. Note that expected male life expectancy at birth in Ethiopia is 64 years (United Nations, 2019) and so we focus on most of this lifespan.

We estimated the potential impact of the tax-induced increase on the price of the most common cigarette brand. Recall, that the reform increases the price of one pack of these cigarettes by 67%. To gauge the probable response of Ethiopian smokers to the excise tax increase, we compiled the total price elasticity of demand for cigarettes identified by a number of previous studies in sub-Saharan Africa (elasticities provided in Table A3) (Berg et al., 2001; Chelwa & van Walbeek, 2019; Economics of Tobacco Control in South Africa Project, 1998; Ho et al., 2017; Kidane et al., 2015; Mukong & Tingum, 2020; Reekie, 1994; Van Walbeek, 1996). These investigations indicated that a 1.00% increase in price reduces total cigarette consumption between 0.30% and 1.73%; the average elasticity estimated from the studies was −0.73 and the interquartile range (IQR) was 0.24. Since price elasticity is expected to vary by income/wealth, with the poor being more price-elastic (Chaloupka et al., 2012; IARC, 2011), we distributed the IQR from these studies around the estimated average elasticity, such that the absolute value of the total price elasticities declined with wealth. Evidence also shows that young individuals are more price-sensitive (Chaloupka et al., 2012; Chaloupka & Wechsler, 1997; IARC, 2011) and so we applied youth modifiers to the price elasticities for the youngest age groups—modifiers of 1.5 for those currently aged 0–24 years. We present the price elasticities computed for the different age and wealth groups in Table 2.

**Table 2:** Assumed total price elasticity of demand for cigarettes across age and wealth quintile categories.

Wealth quintile	Age 15–24 years and future smokers (i.e. individuals <15 years)	Age 25–59 years
Poorest	−1.46	−0.97
Poorer	−1.28	−0.85
Middle	−1.10	−0.73
Richer	−0.92	−0.61
Richest	−0.74	−0.49

### 2.2 Modeling approach

An increase in price is expected to decrease total cigarette consumption by reducing the number of people who smoke—this is the level of smoking participation—and also by reducing consumption among those who continue to smoke. Estimates in the literature suggest that the elasticity of smoking participation is about half the total price elasticity of demand (Becker et al., 1990; Emery et al., 2001; IARC, 2011). The participation elasticity can be used to calculate the number of individuals who quit smoking in the aftermath of the tax increase. Based on earlier estimates of YLG when smoking is ceased at different ages (Doll et al., 2004), we estimated YLG as a function of age at smoking cessation and used predicted YLGs from this model to calculate total YLG over all individuals quitting smoking in each age-wealth grouping.

We calculated tax revenues before and after the tax increase in order to estimate the change in revenues due to the tax reform. Tax revenues at each point in time were the product of the number of smokers at the time, cigarette consumption levels and the amount of the tax. The change in cigarette expenditures for consumers was estimated analogously to the change in tax revenues, except that we used cigarette price as an input for these calculations instead of tax amount.

Using existing estimates of the impact of smoking cessation on tobacco-related premature deaths, we calculated the number of tobacco deaths likely to have occurred in the absence of the tax increase (Doll et al., 2004; Jha et al., 2012), distributed these deaths across the major diseases associated with smoking mortality in Ethiopia (IHME, 2019), and identified whether OOP health costs would be sizeable (in relation to consumption (Kiros et al., 2020)) for those who would have sought treatment for their illnesses prior to dying. We categorized these instances as cases of CHE averted by the tax hike, presenting CHE numbers when OOP costs exceed 10, 25 and 40% of consumption expenditures (see webappendix for further detail).

While the input parameters and strategy described in this section were used for our base-case estimation, we also conducted the following sensitivity analyses. First, we assumed a flat price elasticity of demand for cigarettes across wealth quintiles, using −0.50. This was the mid-point of the price elasticity range identified for low- and middle-income countries (LMICs) in a review by IARC (2011). This figure is lower (in absolute value) than the elasticities for four of the quintiles used in the main analysis. It has been posited that lower price elasticities exist for goods that are available at different prices since consumers can easily move to cheaper brands when prices increase (Hu, Mao, et al., 2016). Accordingly, the flat and relatively lower price elasticity check here could also be interpreted as an examination of the extent to which substitution effects might dampen the impact of the tobacco tax increase. Second, we considered youth to be just as price-elastic as older individuals, not more elastic.

We present all estimates disaggregated by wealth quintile to help gauge whether the policy is progressive or regressive in its effects across the entire socioeconomic spectrum. Costs are expressed in 2019 USD (with 1 USD equivalent to about 29.07 ETB). All computations were conducted using RStudio (version 1.2.5033) and Microsoft Excel (version 16.16.25).

## 3 Results

Table 3 presents the main results of our analysis. We find that Ethiopia's tobacco tax increase could lead to health gains of almost eight million life years. Note that these gains would be experienced by current smokers induced to quit smoking at some point during their lives and might not be realized immediately. The poor are likely to benefit disproportionately: 63% of YLGs would be concentrated among the poorest two quintiles, whereas only 21% would accrue to the richest two quintiles.

**Table 3:** Projected effects of Ethiopia's tobacco tax increase, disaggregated by wealth quintile.

		Wealth quintile				
Outcome	Total	Poorest	Poorer	Middle	Richer	Richest
Years of life gained	7,831,000	2,478,000	2,466,000	1,260,000	624,000	1,003,000
Change in annual tax revenues^a^						
In year 1	26,173,000	2,218,000	3,432,000	4,545,000	2,361,000	13,617,000
In year 10	19,707,000	461,000	2,360,000	4,340,000	1,627,000	10,919,000
Change in annual expenditures on cigarettes^a^						
In year 1	−18,544,000	−10,483,000	−8,013,000	−2,952,000	−338,000	3,242,000
In year 10	−36,477,000	−15,351,000	−18,615,000	−3,558,000	−615,000	1,662,000
Catastrophic health expenditures averted						
At 10% threshold	173,000	62,000	61,000	29,000	10,000	12,000
At 25% threshold	76,000	35,000	21,000	11,000	6,000	4,000
At 40% threshold	55,000	21,000	20,000	10,000	3,000	0

All estimates are rounded to the nearest 1,000.

a In 2019 USD.

In terms of tax revenues, the reform could increase total revenues by USD26 million in year one. More than half this increase would be borne by individuals in the richest quintile. Since higher taxes are likely to deter smoking initiation among younger individuals, fewer people would smoke in the future and so the growth in revenues would decline in the long run. Note though that the government would collect higher revenues than before the tax increase even 10 years later (greater by USD20 million). Given the higher price elasticity of poorer individuals, more poor individuals can be expected to quit smoking after the reform and therefore the poorest quintile would contribute the least to the future increases in tax revenues.

Next, we show that aggregate cigarette expenditures for consumers would likely decline after the tax reform. This is due to the fact that some previous smokers will quit smoking and those who continue smoking are likely to smoke less frequently after the tax hike. Here again, the bulk of the averted expenditures would accrue to poorer quintiles. Only the richest quintile would see cigarette expenditures go up after the tax increase.

Finally, we estimate that the tobacco tax hike, by reducing smoking-related mortality and the medical spending leading up to these deaths, would avert a significant number of CHE cases: 173,000, 76,000 and 55,000 cases at 10, 25 and 40% thresholds of total consumption expenditures. The poorest quintile is likely to bear the greatest share of these gains. These CHE benefits will be realized at some point during the lifetime of smokers induced to quit smoking by the tax reform.

Next, we present results from two sensitivity analyses (Table 4). First, when we assigned all quintiles a flat price elasticity of demand for cigarettes of −0.50, we found that Ethiopia's tax hike could lead to health gains of about five million life years. As in the main results, the combined benefits to the bottom two quintiles would constitute more than half of the total YLGs. Under the flat elasticity scenario, individuals in all but one group would be less price responsive than in the main analysis. There would thus be lower reductions in smoking. Accordingly, tax revenues in the year after the reform would increase more than in the base case—an increase of USD40 million. Here too, the richest quintile would pay the highest share of the net change in tax revenues. In contrast to the base case, the change in cigarette expenditures in the year after the tax increase would be positive—this is because more individuals would continue to smoke. Furthermore, fewer CHE cases would be averted: 108,000 CHE cases at the 10% threshold.

**Table 4:** Sensitivity analyses - Projected effects of Ethiopia's tobacco tax increase, disaggregated by wealth quintile.

		Wealth quintile				
Outcome	Total	Poorest	Poorer	Middle	Richer	Richest
*Flat price elasticity of demand across wealth quintiles of -0.50*						
Years of life gained	5,126,000	1,277,000	1,450,000	863,000	511,000	1,024,000
Change in annual tax revenues^a^	40,073,000	8,574,000	8,283,000	6,956,000	2,855,000	13,405,000
Change in annual expenditure on cigarettes^a^	8,387,000	1,832,000	1,386,000	1,720,000	618,000	2,832,000
Catastrophic health expenditures averted						
At 10% threshold	108,000	32,000	36,000	20,000	8,000	12,000
At 25% threshold	46,000	18,000	12,000	7,000	5,000	4,000
At 40% threshold	32,000	11,000	12,000	7,000	2,000	0
*Youth just as price elastic as older individuals*						
Years of life gained	6,455,000	2,053,000	1,942,000	1,098,000	511,000	852,000
Change in annual tax revenues^a^	28,196,000	2,746,000	4,269,000	4,662,000	2,466,000	14,052,000
Change in annual expenditure on cigarettes^a^	−14,624,000	−9,460,000	−6,391,000	−2,724,000	−135,000	4,087,000
Catastrophic health expenditures averted						
At 10% threshold	143,000	51,000	48,000	25,000	8,000	10,000
At 25% threshold	63,000	29,000	17,000	10,000	5,000	3,000
At 40% threshold	45,000	18,000	15,000	9,000	2,000	0

All values are presented in 2019 USD.

All estimates are rounded to the nearest 1,000.

a For the first year after the tax increase.

Second, upon assuming that price elasticities do not vary by age, we estimate gains of around six million life years. Even in this case, the greatest share would occur amidst the poorest two quintiles. Tax revenues would increase by USD28 million in the year after the tax hike; half of this increase would be borne by the richest quintile. Overall cigarette expenditures would fall starting in the first year and the poorest quintile would experience the greatest decline; the richest quintile would, however, spend an extra USD4 million in year one. Finally, the number of CHE cases averted in this scenario would lie in between the estimates identified for the main scenario and for the flat price elasticity scenario.

Combining the results from the main analysis and sensitivity analyses suggests that Ethiopia's tobacco tax reform could save roughly between five and eight million life years within the male population. In the first year after the tax hike, increases in aggregate revenues could range between $26 and $40 million. Furthermore, tobacco-related disease reductions stemming from smoking cessation due to the tax hike could avert between 108,000 and 173,000 CHE cases (at a 10% threshold). These ranges imply that while there is some uncertainty about the magnitude of the potential gains of this tobacco policy change, the health and financial impacts are likely to be sizeable. Also, the broad distribution of impacts across quintiles is consistent in all the scenarios investigated: the poor would benefit disproportionately. This suggests that the effects of Ethiopia's tobacco tax reform would likely be progressive.

## 4 Discussion

We modeled in this analysis the potential future impacts of the Ethiopian government's most recent effort to regulate smoking practices and tobacco prices: tobacco taxes were increased in early 2020 and accordingly, the price of cigarettes increased by 67% (Stoklosa, 2020). In our investigation, we document potential effects on one health outcome (YLGs among those induced to cease smoking) and three financial outcomes (tax revenues collected, cigarette expenditures faced by consumers, and CHE cases averted).

Our results indicate that increased cigarette taxes in Ethiopia could lead to substantial smoking cessation, thereby saving millions of life years—almost eight million over the lifespan of current (and future) smokers induced to quit by the tax hike. Importantly, the bulk of these benefits would likely accrue to the poorest, with those in the bottom two quintiles experiencing around 60% of the gains. The tax hike would boost revenues, with a USD26 million increase in tax revenues in the first year after the reform. Note that this represents about 7% of health expenditures dedicated to non-communicable diseases (NCDs) in Ethiopia, which were about $4 per capita in 2017 (Federal Democratic Republic of Ethiopia Ministry of Health, 2019a) for a population of just over 100 million (World Bank, 2020). Such boosts in revenues could be used to finance universal health coverage efforts, NCD interventions and other pro-poor investments, thereby potentially reinforcing the progressivity of the tobacco tax increase. It is important to note that the approach of earmarking “sin” taxes to support public health efforts has been used in countries like Thailand and the Philippines (Alebachew & Mitiku, 2019). We show that while the Ethiopian government would continue to collect higher revenues in the future (relative to the pre-tax increase period), the increment would decline over time since the high taxes will deter smoking initiation among young individuals in the future. What our results highlight is that the primary value of tobacco tax hikes in relatively low prevalence settings lies in their capacity to generate substantial health benefits by deterring smoking initiation among future generations. Finally, our results point to the potential that the reform has to enhance consumers’ financial wellbeing, notably by significantly reducing the potentially catastrophic expenditures that arise due to the OOP treatment costs associated with tobacco-related diseases. On this indicator too, the poorer quintiles of the population are expected to benefit disproportionately. This is a substantial contribution given that OOP payments finance about 70% of NCD expenditures in Ethiopia, thereby imposing a high risk of catastrophic expenditures (Ethiopia NCDI Commission, 2018).

This analysis makes several valuable contributions to the literature. By studying the implications of tobacco taxation in Ethiopia, we contribute to the very sparse literature on this topic in sub-Saharan Africa: the majority of the studies in this region have focused on a single country—South Africa (Berg & Kaempfer, 2001; Economics of Tobacco Control in South Africa Project, 1998; Mukong, 2020; Reekie, 1994; Van Walbeek, 1996). Furthermore, we point to the sizeable mortality reduction benefits that are likely to stem from tobacco tax increases in countries that currently have low smoking prevalence. In such settings, tax hikes can be a powerful protective strategy against the future health costs and death toll of smoking. Tobacco regulation is particularly needed to safeguard public health in low- and middle-income countries (LMICs) since the tobacco industry is increasingly using aggressive marketing strategies to tap into new markets in Africa (Erku & Tesfaye, 2019; Gilmore et al., 2015; Husain et al., 2016). The extent to which external tobacco players are looking to engage with African countries is best exemplified for the study setting by the 2016 takeover of almost half of the premier Ethiopian tobacco company's shares by JTI, a global tobacco company (Endeshaw, 2016; Erku & Tesfaye, 2019). As we demonstrate, Ethiopia's tobacco tax hike could achieve substantial benefits even under these circumstances and the potential effects could be higher if the government were to additionally leverage all the components of WHO's MPOWER package (WHO, 2021a): for example, if it implemented tobacco tax reform in conjunction with the provision of comprehensive tobacco cessation services.

Our analysis does have a number of important limitations. First, we were not able to estimate the price elasticity of demand for tobacco products specifically for Ethiopia. Instead, we based the elasticities that we used on those identified in similar settings, ensuring that the numbers demonstrated the pattern of variation across age and income documented in the literature (Chaloupka et al., 2012; Chaloupka & Wechsler, 1997; IARC, 2011). It is worth pointing out that the average elasticity we pulled from various studies (−0.73) is within the range identified by IARC for LMICs (−0.20 to −0.80) (IARC, 2011). We also conducted several sensitivity analyses using alternative price elasticities to gauge the possible range within which future impacts on the variables of interest are likely to lie. Second, since we estimated population distribution and smoking prevalence across age and wealth categories from the DHS, it is important to point out that samples within these categories invariably shape our parameter values. Specifically, some of the variation in parameters could be driven by small sample sizes within cells. Third, we were limited in our ability to account for the dynamic ways in which producers and/or consumers might respond to tobacco taxes. To illustrate, the increase in tax could be absorbed by the tobacco industry at least in part, which has been observed to occur in a few settings (Hu, Zhang, & Zheng, 2016; Ross et al., 2017). If this were to transpire in Ethiopia, the future effects would be lower than those that we document here. Consumers too might move to cheaper cigarette brands or other tobacco products that are taxed to a lower extent. Such substitution effects could mute the effectiveness of the tax increase in Ethiopia, effects that are likely to be particularly prevalent in the rural areas of the country where local tobacco products are sold openly and are not subject to strong taxation enforcement (Guliani et al., 2019). It is important to note here though that the 2020 tobacco reform introduced a specific excise tax, which reduces the variation in prices across different brands of a product (Jha & Peto, 2014; White et al., 2013). There is, however, scope to further limit substitution efforts by increasing the tobacco excise tax even more. Fourth, our model did not account for the potential effects of the illicit cigarette trade. If illicit cigarettes are available to consumers at prices that are lower than those that are subject to the tax increase, the potential impact of the tax reform would be lower than the estimates that we provide here. While the tobacco industry tends to argue against tax increases by pointing to the potential they have to increase the size of the illicit cigarette market, a recent investigation into the scale of these operations in Ethiopia shows that illicit cigarette consumption is low in most of the country and that the overall tobacco market share captured by illicit products in Ethiopia is likely to be significantly lower than claims made by the tobacco industry (Dauchy & Ross, 2022). Since illicit cigarettes account for a high share of the cigarette market in only certain areas adjacent to borders (accounting for just 4% in inland areas), it is likely that the illicit market in Ethiopia is driven by poor tobacco control and greater access to cheap substitutes in border regions rather than tax policy (Dauchy & Ross, 2022). Fifth, we were limited by our ability to model the potential implications of varying levels of addiction. If a significant proportion of Ethiopia's current smokers are highly addicted to tobacco, they would not be very responsive to the tax increase and more individuals would continue to smoke in the aftermath of the policy change. In a check (see webappendix Table A5), we do however demonstrate that even if the poor in Ethiopia were potentially more addicted to tobacco than their richer counterparts (hence less price responsive than assumed in the main analysis), they would still be expected to benefit from the tobacco tax reform. Sixth, factors besides wealth and income should be examined in order to assess whether a policy is regressive or progressive in its effects, such as considerations related to education and residence (urban versus rural). Our findings are unable to speak to how future impacts might vary along such non-wealth criteria. We estimate and present potential consequences for Ethiopian men as a whole, not accounting for heterogeneities in tobacco use patterns and other input parameters across groups defined by characteristics such as region of residence even though such variations could be large (Erku & Tesfaye, 2019). Since we do not factor these differences into our analysis, we are unable to provide a sense of how different groups (e.g. across different regions) in Ethiopia might fare in the aftermath of the tax increase. While we excluded women from our main analysis due to low levels of smoking prevalence (Central Statistical Agency (CSA) Ethiopia and ICF, 2016), we anticipate that women would also likely benefit from the tobacco tax reform (Tables A6-A7). Seventh, it is important to acknowledge that the tobacco tax increase could have economic costs that are not quantified in this analysis. For example, reductions in tobacco sales due to the reform could lead to job losses among those involved with tobacco production and sales. Research in different settings, however, shows that tax increases do not have net negative impacts on jobs (Chaloupka et al., 2019). Finally, the health impacts we highlight (i.e. the mortality reduction impacts) are likely to be underestimates. We quantify the potential health benefits only for the individuals who quit smoking in response to the reform. Note that individuals who continue to smoke after the tax hike will likely reduce total cigarette consumption—for example, by moving from daily to non-daily smoking—and might therefore also experience a decline in their risk of mortality (Inoue-Choi et al., 2020). Furthermore, by limiting our attention to mortality, we are not accounting for reductions in morbidity, including those emerging due to decreases in exposure to secondhand smoke. Due to these factors, our quantification of the financial costs of tobacco-driven poor health are also likely to underestimate the projected consequences of the reform. Future studies could fill some of these research gaps by using alternative sources of data (e.g. STEPS survey of NCDs (Ethiopian Public Health Institute, 2016)).

The 2020 tobacco tax change prescribed a sizeable increase in the price of cigarettes in Ethiopia—while taxes used to account for less than 20% of the price (WHO, 2019), they now account for about 50% of the price. However, it is important to point out that this level of tobacco taxes still falls short of the WHO recommendation that taxes constitute at least 70% of the retail price of tobacco (WHO, 2011). Increases in government revenues from additional tobacco tax hikes (coupled with other “sin” taxes on products like alcohol and sugary drinks) (Miracolo et al., 2021; Wright et al., 2017) could prove critical for maintaining government revenue flows during economic downturns in Ethiopia, including the current global economic crisis due to COVID-19 (Dione, 2020).

## Ethical statement

We, the authors, declare that we have no conflicts of interest. Furthermore, we have no financial disclosures.

## Financial disclosure

None.

## Author statement

Averi Chakrabarti: Conceptualization, Methodology, Identifying Relevant Input Parameters, Formal Analysis, Writing – Original Draft Preparation.

Solomon Tessema Memirie: Identifying Relevant Input Parameters, Writing – Review and Editing.

Seblewongel Yigletu: Identifying Relevant Input Parameters, Writing – Review and Editing.

Mizan Kiros Mirutse: Identifying Relevant Input Parameters, Writing – Review and Editing.

Stéphane Verguet: Conceptualization, Methodology, Supervision, Identifying Relevant Input Parameters, Writing – Review and Editing.

## Declaration of competing interest

None.
