# Supplementary material for: The potential distributional health and financial benefits of increased tobacco taxes in Ethiopia: Findings from a modeling study
Source: SSM Popul Health. 2022 Apr 14;18:101097. doi: 10.1016/j.ssmph.2022.101097 (PMC9127671; doi:10.1016/j.ssmph.2022.101097)
Supplement: Multimedia component 1 [file mmc1.pdf]

Supplementary webappendix

The potential distributional health and financial benefits of increased tobacco taxes in Ethiopia: Findings from a modeling study

---

1. <u>Tobacco regulation in Ethiopia</u>

Table A1 lists tobacco regulation policies enacted in Ethiopia and the corresponding years of implementation.

Table A1: Tobacco regulation in Ethiopia

Year	*Policy*
2004	Ethiopia signs the World Health Organization (WHO) Framework Convention on Tobacco Control (FCTC).
2009	The Ethiopian Food, Medicine and Healthcare Administration and Control Authority (FMHACA) is authorized to regulate the tobacco industry in Ethiopia (including tobacco manufacturing, packaging and sales).
2012	Tobacco advertisements are prohibited.
2013	FMHACA receives the mandate to impose various tobacco restrictions.
2014	Ethiopia ratifies the WHO FCTC.
2015	FMHACA issues a National Tobacco Control Directive to introduce several tobacco control regulations such as bans on tobacco sales to minors and prohibitions on the sale of flavored tobacco products.
2017	The National Tobacco Control Coordinating Committee (NTCCC), which was set up at the federal level to oversee tobacco control efforts, releases the draft of Ethiopia's first National Tobacco Control Strategic Plan covering the years between 2017 and 2020. This plan sets out the objectives to be achieved in pursuance of the WHO FCTC.
2019	Tobacco control legislation is enacted to mandate smoke-free public spaces, bans on tobacco advertisements, restrictions on the sale of tobacco products and the inclusion of warning labels on tobacco products.
2020	Tobacco excise taxes are increased.

Sources: Erku and Tesfaye, 2019; Federal Democratic Republic of Ethiopia, 2019; Marquez, 2019; Stoklosa, 2020.

2. Data inputs

Table A2: Detailed input parameters used by age and wealth

	Poorest	Poorer	Middle	Richer	Richest
Panel A: Male population distribution (proportions)					
0-4	0.017	0.017	0.015	0.013	0.010
5-9	0.020	0.018	0.016	0.016	0.012
10-14	0.017	0.017	0.017	0.017	0.013
15-19	0.007	0.007	0.008	0.010	0.010
20-24	0.004	0.006	0.005	0.007	0.007
25-29	0.005	0.006	0.006	0.006	0.010
30-34	0.004	0.005	0.005	0.005	0.007
35-39	0.004	0.004	0.005	0.005	0.006
40-44	0.004	0.004	0.004	0.004	0.004
45-49	0.003	0.003	0.003	0.003	0.003
50-54	0.002	0.002	0.002	0.002	0.002
55-59	0.001	0.001	0.002	0.002	0.002
Panel B: Male smoking prevalence (proportions)					
	Poorest	Poorer	Middle	Richer	Richest
15-19	0.036	0.002	0.001	0.007	0.012
20-24	0.042	0.109	0.043	0.028	0.059
25-29	0.172	0.063	0.090	0.042	0.092
30-34	0.205	0.174	0.142	0.053	0.066
35-39	0.181	0.246	0.174	0.060	0.123
40-44	0.229	0.175	0.131	0.074	0.222
45-49	0.136	0.122	0.240	0.050	0.116
50-54	0.242	0.136	0.164	0.186	0.175
55-59	0.113	0.014	0.011	0.057	0.156
Panel C: Average per capita daily cigarette consumption					
	Poorest	Poorer	Middle	Richer	Richest
15-19	2.24	9.40	1.15	0.14	0.33
20-24	7.29	4.75	2.42	2.83	6.98
25-29	3.35	2.31	3.53	3.21	11.37
30-34	3.41	6.81	1.53	2.74	5.27
35-39	3.52	6.44	3.31	4.40	3.22
40-44	4.66	3.07	3.37	2.38	6.19
45-49	5.59	3.27	10.84	3.04	3.44
50-54	8.63	5.10	2.25	5.63	18.63
55-59	5.21	3.79	11.68	3.65	6.36

Source: The 2016 Ethiopian Demographic and Health Survey (DHS). Sample weights used for all statistics derived from the DHS.

Note: The DHS did not survey boys below the age of 15 years and so we do not have data on smoking prevalence and total cigarette consumption for these groups. We assumed that children and youth below 15 years are yet to initiate smoking but that in the absence of the tax increase, their future tobacco use patterns would have been akin to those currently observed amongst individuals aged 15-24 years.

3. Modeling approach

In this section, we describe in detail the considerations behind the selection of critical input parameters (e.g. price elasticity of demand for tobacco) and the different steps underlying our modeling approach. The approach we use builds on that adopted by past analyses (Verguet et al., 2015; Postolovska et al., 2018; James et al., 2019).

3.1. Baseline cigarette consumption

To estimate pre-tax smoking prevalence and cigarette consumption levels, we used data from the Demographic and Health Survey (DHS) conducted in Ethiopia in 2016 (Central Statistical Agency and ICF, 2016). Note that since this survey was administered prior to the tobacco tax increase, any smoking-related data collected could be used to proxy pre-policy or baseline smoking levels. Since we conducted our analysis only for men, we utilized smoking data collected through the adult male questionnaire of the DHS. We categorized smokers as those who smoked daily or occasionally, and estimated the proportion of men in each age and wealth category who smoked.

In the DHS, smokers were asked about their level of cigarette consumption—daily smokers reported the number of cigarettes smoked per day and occasional smokers reported the number of cigarettes smoked per week. We computed the daily cigarette consumption of occasional smokers by dividing by seven the weekly count that these individuals reported. Using the daily consumption figures for regular and occasional smokers, we identified the average number of cigarettes smoked per day by smokers in each age-wealth group. We then converted these mean daily cigarette

consumption numbers to the average number of annual cigarette packs consumed by smokers in each age (denoted by a)-wealth grouping (denoted by i):

$$C_1(a,i) = \frac{Cig(a,i) \times 365.25}{20} \quad , \qquad (1)$$

where C_1 is the typical annual cigarette pack consumption per capita in each age-wealth category in period 1 (i.e. the pre-tax increase period). Cig is the average number of cigarettes consumed per day by a smoker in the group; the number of cigarettes in a pack is assumed to be 20.

Note, that we only used data on the consumption of manufactured cigarettes. Respondents separately reported consumption frequencies for hand-rolled cigarettes. The average number of manufactured cigarettes smoked by smokers per day is 5; the corresponding number for hand-rolled cigarettes is about 0.5. The latter type of cigarettes tends to be produced largely by the informal sector in countries around the world, and are usually hard to regulate and tax (Young et al., 2008; Laxminarayan and Deolalikar, 2004; Joossens et al., 2014; Shimkhada and Peabody, 2003). In fact, Ethiopia's tobacco laws have typically targeted manufactured cigarettes (Dauchy and Ross, 2022). Accordingly, we dropped hand-rolled cigarettes when quantifying cigarette consumption in our analyses.

3.2. *Number of smokers at baseline*

To estimate the number of male smokers in Ethiopia at baseline, we combined population proportions and smoking prevalence from the DHS with total country population from the World

Development Indicators (the 2016 figure) (World Bank, 2020). The baseline number of male smokers is given by:

$$S_1(a,i) = Pop(a,i) \times TP \times Prev(a,i) \quad . \quad (2)$$

Here, Pop is the proportion of the total country population that men in an age-wealth group (a,i) constitute. TP is the total population of the country and $Prev$ is the proportion of each age-wealth group (a,i) of men that smokes.

3.3. Total price elasticities of demand for cigarettes

Table A3: Price elasticities of demand for cigarettes in sub-Saharan African settings

Country	Price elasticity	Sources	Notes
36 African countries	-0.56	Ho et al., 2017	We used the price elasticity identified by the authors for the low-income countries using a fixed effects model (see p. 905).
South Africa	-0.88	Reekie, 1994	
South Africa	-0.66	van Walbeek, 1996	The author found that short-run price elasticities lie between -0.32 and -0.99. We used the mid-point of these figures.
South Africa	-0.58	Economics of Tobacco Control in South Africa Project, 1998	The study showed that short-run elasticities lie between -0.57 and -0.59. We used the mid-point of these figures.
South Africa	-0.80	Berg and Kaempfer, 2001	We used the price elasticity identified for Black individuals (see p. 1171).
South Africa	-0.56	Mukong and Tingum, 2020	This study indicated a -0.43 price elasticity for economy brands and a -0.69 price elasticity for mid-price brands. We used the mid-point of these figures.
Tanzania	-1.73	Kidane et al., 2015	
Uganda	-0.30	Chelwa and van Walbeek, 2019	The authors found price elasticities to range between -0.26 and -0.33. We used the mid-point of these figures.
Zimbabwe	-0.52	Economics of Tobacco Control in South Africa Project, 1998	This is the short-run elasticity.

The nine studies above had an average price elasticity (or μ) of -0.73, a median of -0.58, an interquartile range (IQR) of 0.24, and a standard deviation (SD) of 0.41. The poor are expected to be more responsive to changes in price (IARC, 2011; Chaloupka et al., 2012), and therefore we distributed the IQR around the average μ to obtain price elasticities that decline in absolute value with wealth. Specifically, we used the following formulae to identify total price elasticities (or PE) for the different quintiles:

$$PE_1 = \mu - IQR = -0.97,$$

$$PE_2 = \mu - \frac{IQR}{2} = -0.85,$$

$$PE_3 = \mu = -0.73,$$

$$PE_4 = \mu + \frac{IQR}{2} = -0.61,$$

$$PE_5 = \mu + IQR = -0.49. \tag{3}$$

Since youth are more price sensitive than older individuals (Chaloupka and Wechsler, 1997; IARC, 2011; Chaloupka et al., 2012), we further adjusted these price elasticities for the younger age groups, applying modifiers of 1.5 for those aged 0-24 years.

3.4. *Years of life gained*

The elasticity of smoking participation was set to half the price elasticity of demand (Becker et al., 1990; Emery et al., 2001; IARC, 2011). Therefore, the number of baseline smokers in each age-wealth grouping (a, i) who quit in the aftermath of the tax increase was estimated with the following:

$$Q(a,i) = -S_1(a,i) \times \frac{1}{2} \times \varepsilon(a,i) \times \frac{\Delta p}{p} \quad , \quad (4)$$

where $\varepsilon(a,i)$ is the total price elasticity of demand for cigarettes in age group a and quintile i, p is baseline price of cigarettes and Δp is the change in this price after the tax increase.

Based on the findings of Doll et al. (2004) that showed that smoking cessation at ages $a = 30$, 40, 50 and 60 years would lead to about 10, 9, 6 and 3 years of life gained (YLGs) respectively, we modeled YLG when quitting smoking at different ages using the following model:

$$YLG(a) \sim \beta_0 + \beta_1 a \quad . \quad (5)$$

Next, we predicted YLG for the different age groups (using roughly the mid-point for each group, for example, 12.5 years for those 10-14 years) or $YLG(a)$. We capped this variable at 10 years, the maximum gain in the aforementioned study (Doll et al., 2004):

$$YLG(a) = \widehat{\beta_0} + \widehat{\beta_1} a \quad . \quad (6)$$

Subsequently, we calculated YLG for all age-wealth groups $TYLG$ with the following product:

$$TYLG(a,i) = Q(a,i) \times YLG(a) \quad . \quad (7)$$

Note, these are gains that would be experienced by current smokers at some point during their entire lifetime, not necessarily in the immediate future.

3.5. *Change in tax revenues and cigarette expenditures*

The change in tax revenues due to the reform can be estimated with:

$$\Delta Tax\_revenue = (TK_2 \times t_2) - (TK_1 \times t_1) \quad , \qquad (8)$$

where $TK_1 = \sum C_1 S_1$ is total cigarette consumption before the reform by men in all age-wealth groupings. Total consumption after the reform (TK_2) would be the sum across age group a and quintile i of $TK_1(a, i) \times (1 + \varepsilon(a, i) \times \frac{\Delta p}{p})$. $t_1$and t_2 are tax amounts per cigarette pack before and after reform, respectively.

The last outcome that we probed, the change in cigarette expenditures, was estimated analogously:

$$\Delta Cig\_expenditures = (TK_2 \times p_2) - (TK_1 \times p_1) \quad , \quad (9)$$

where $p_1$and p_2 are price per cigarette pack before and after the tax increase, respectively. All other variables are as described previously.

We presented changes in tax revenues and cigarette expenditures at two points in time—1 and 10 years after the tax is implemented. In the immediate aftermath of the tax hike—that is, in year 1—we assumed that individuals aged 15-59 years would comprise the population of interest. In other words, these groups would contain individuals responsible for all cigarette purchases (i.e. all smokers) at this time. The assumption was that younger individuals would not have initiated

smoking yet. We thus estimated equations (8) and (9) only for individuals 15-59 years in year 1. In year 10, the age groups containing all smokers (those who pay tobacco taxes and incur cigarette expenditures) would be those who are currently between the ages of 5 and 49 years. Those aged 5-14 years would presumably have started smoking by this time. Additionally, we ensure that the oldest individual at year 10 is the same age as the oldest individual in the sample in year 1, in other words, 59 years in year 10 or 49 years currently.

3.6. Financial risk protection

For the financial risk protection (FRP) calculations, we first estimated the number of deaths averted by the tax increase. Note that as in the estimation of total YLGs due to the tax hike, we did not limit our attention to a specific time span but accounted for all deaths averted sometime during the lifespan of the population currently aged 0-59 years, irrespective of when they are expected to occur. The starting point for these calculations were the following considerations: (1) about $\frac{1}{2}$ of persistent smokers die of smoking-related illness (Doll et al., 2004), and (2) the likelihood of smoking-related premature death falls by 97, 85 and 75% for individuals quitting at ages 15-24, 25-44 and 45-64 years, respectively (Jha et al., 2012). Accordingly, deaths averted ($Total\_deaths\_averted$) for each group (*a, i*) would be given by:

$$Total\_deaths\_averted(a,i) = Q(a,i) \times \frac{1}{2} \times Prob\_decline(a) \quad , \qquad (10)$$

where, as described above, $Q(a,i)$ represents the number of smokers in each age-wealth group (*a, i*) who quit smoking in the aftermath of the tax increase, and $Prob\_decline(a)$ is 0.97, 0.85 and 0.75 for the 15-24, 25-44 and 45-64 age groups, respectively.

Next, we sourced the number of deaths attributable to tobacco for Ethiopia in 2019 (the last year for which these data were available) from the Global Burden of Disease (GBD) study (IHME, 2019), focusing on deaths due to behavioral factors. Tobacco deaths are further subdivided into deaths due to smoking, secondhand smoke, and chewing tobacco. We used data only for the first category. The following diseases accounted for 80% of all smoking deaths in Ethiopia: tuberculosis (TB) (17%), neoplasms (15%), cardiovascular disease (CVD) (27%) and chronic obstructive pulmonary disease (COPD) (20%). Accordingly, we focused on just these tobacco-related diseases in estimating the OOP costs and associated financial risk (i.e. the likelihood of catastrophic health expenditures (CHE)) averted by tax-induced smoking reductions in Ethiopia. Essentially, we gauged the extent to which the reductions in smoking would be able to avert OOP treatment costs by preventing deaths from the four main tobacco-related diseases.

For the estimated deaths averted in each age-wealth category, we portioned out deaths to the four diseases of interest. In doing this, we inflated the estimated proportions of smoking deaths caused by each of the four diseases of interest by $\frac{1}{0.80}$ in order to account for all tobacco deaths averted by the tax (this adjustment is needed since the diseases of interest account for 80% of tobacco deaths). Specifically, the deaths averted due to a specific disease *d* were estimated as:

$$Deaths\_averted_d = Total\_deaths\_averted \times s \times \frac{1}{0.80} \quad , \qquad (11)$$

where s = 0.17, 0.15, 0.27 and 0.20 for TB, neoplasm, CVD and COPD, respectively (as indicated by the GBD study estimates) (IHME, 2019).

We derived estimates for OOP costs due to disease treatment using inputs from the published literature (Hailu et al., 2013; Tolla et al., 2017; Ethiopia NCDI Commission, 2018; Assebe et al., 2020; Watkins et al., 2020). Some of these sources reported estimates by income quintile. For OOP costs for which such a gradient was not available, we assumed that all wealth groups incurred the same costs. The cost estimates were annual measures (the COPD costs, however, were approximate unit costs or costs per COPD case) and were converted into 2019 USD.

Additionally, we made assumptions about care-seeking proportions for each disease of interest. For CVD, we used numbers available for hypertension. Of all individuals with high blood pressure in Ethiopia, 40% are diagnosed and of these, 28% take medication (Ethiopia NCDI Commission, 2018). Accordingly, we assumed that $0.40 \times 0.28 = 0.11$ proportion of individuals with CVD seek care. Currently in Ethiopia, 71% of TB cases are detected (Fekadu et al., 2017), and we assumed that all detected cases would seek at least some care (note that despite public financing of TB care in Ethiopia, patients continue to bear OOP costs (Assebe et al., 2020)). The cancer most likely to arise from smoking is lung cancer (Gandini et al., 2008) and since lung cancer and COPD can have symptoms consistent with those related to TB, we used the same care-seeking proportion (0.71) for these diseases. Given limited information on the socio-economic gradient in care-seeking for these diseases, we used the same numbers across quintiles. Table A4 below reports the OOP and care-seeking figures used in the analysis.

Table A4: Input data for out-of-pocket (OOP) treatment costs and care-seeking behavior

	Wealth quintile				
	**Poorest**	**Poorer**	**Middle**	**Richer**	**Richest**
**OOP treatment costs** ($)					
Tuberculosis (TB)	50	79	106	140	202
Neoplasm	459	459	459	459	459
Cardiovascular disease (CVD)	110	237	343	501	666
Chronic obstructive pulmonary disease (COPD)	86	86	86	86	86
**Care-seeking proportion**					
Tuberculosis (TB)	0.71	0.71	0.71	0.71	0.71
Neoplasm	0.71	0.71	0.71	0.71	0.71
CVD	0.11	0.11	0.11	0.11	0.11
COPD	0.71	0.71	0.71	0.71	0.71

Notes:

TB: We used mean total costs for TB from Table 3 in Assebe et al., 2020. Care-seeking proportion for this disease is from Fekadu et al., 2017.

Neoplasm: Hailu and Mariam (2013) reports costs for cervical cancer. We computed costs from Tables 2-4, summarizing median outpatient (direct and indirect) and inpatient (direct and indirect) expenditures.

CVD: We use data reported by Tolla et al., (2017) in Table A.2.2.1 in the supplementary annex. We used mean costs. Care-seeking proportion for this disease is from Ethiopia NCDI Commission, 2018.

COPD: We averaged low-income country costs reported in Watkins et al. (2020) for the following two diseases: 'Management of acute exacerbations of asthma and COPD using systemic steroids, inhaled beta-agonists, and, if indicated, oral antibiotics and oxygen therapy' and 'Low-dose inhaled corticosteroids and bronchodilators for asthma and for selected patients with COPD'. Subsequently, we used 0.68 of this average since OOP expenditures were estimated to roughly constitute this proportion of total non-communicable disease expenditures in Ethiopia (Ethiopia NCDI Commission, 2018).

All values are presented in 2019 USD. Variation in OOP costs for neoplasms and COPD were not available for different wealth quintiles.

Based on the care-seeking proportion data, we identified the subset of individuals in each quintile who would have died from a smoking-related disease *and* would have sought care for that disease. We assumed only these individuals would have incurred OOP treatment costs and would have been at risk for CHE in the absence of the tax reform (this is a conservative assumption):

$$Individuals\_at\_risk_d = Deaths\_averted_d \times Care\_seeking_d \quad . \ (12)$$

$Care\_seeking_d$ is the proportion of individuals likely to seek care for disease d. All subsequent calculations are conducted only for the individuals at risk.

Next, we assigned per capita annual expenditures (or per adult equivalent annual consumption ($cons$)) (Kiros et al., 2020) for the individuals at risk in each quintile based on the distribution of consumption expenditures observed in the 2015/16 Ethiopian Household Consumption and Expenditure Survey (Federal Democratic Republic of Ethiopia, 2016). We then estimated the share of per capita consumption constituted by the OOP treatment costs that would have been borne by each individual at risk. If the share was higher than a 10% threshold, the individual would have experienced CHE:

$$CHE = 0 \quad if \ \frac{OOP_d}{Cons} \leq 0.10\,; \qquad CHE \ = 1 \quad if \ \frac{OOP_d}{Cons} > 0.10\ . \quad (13)$$

The sum of all individuals at risk for CHE in each age-wealth category represents the CHE cases avoided in that group upon the increase of tobacco taxes. We also computed these CHE numbers for alternative thresholds: 25% and 40% of consumption expenditures.

Note that we assumed that an individual who would have died due to one of the smoking-related diseases would only incur one year of OOP costs. In other words, we considered these costs to be one-time costs. While this assumption is likely to be an underestimate of the actual costs that might be borne, it helps illustrate, conservatively, the minimum level of financial protection afforded by Ethiopia's tobacco tax increase.

We also acknowledge that our use of current costs and consumption figures implies that in the absence of the tax increase, deaths linked to existing levels of smoking would occur in the near future. These deaths could occur many years later when OOP treatment costs, incomes and consumption levels are significantly different, but attempts to model such changes would introduce complications into our estimation process without providing additional insight and without being empirically grounded. Also, any potential future changes to OOP costs might track changes to consumptions (for example, due to inflation).

4. **Additional results**

In the sensitivity analysis below, we assumed that individuals in the poorest quintiles had greater levels of tobacco addiction than their richer counterparts. To capture the potential implications of addiction, we considered the price elasticities for these groups to be two-thirds of those used in the main analysis. Akin to the main results, these estimates indicate that the poor would likely benefit disproportionately from the tobacco tax reform on virtually all the studied outcomes.

Table A5: Projected effects of Ethiopia's tobacco tax increase, disaggregated by wealth quintile - accounting for potentially lower price responsiveness among poorest two quintiles due to higher levels of addiction

	Wealth quintile					
Outcome	Total	Poorest	Poorer	Middle	Richer	Richest
Years of life gained	6,183,000	1,652,000	1,644,000	1,260,000	624,000	1,003,000
Change in annual tax revenues[1]						
In year 1	34,472,000	6,591,000	7,359,000	4,545,000	2,361,000	13,617,000
Change in annual expenditures on cigarettes[1]						
In year 1	-2,464,000	-2,011,000	-405,000	-2,952,000	-338,000	3,242,000
Catastrophic health expenditures averted						
At 10% threshold	132,000	41,000	40,000	29,000	10,000	12,000
At 25% threshold	58,000	23,000	14,000	11,000	6,000	4,000
At 40% threshold	41,000	14,000	13,000	10,000	3,000	0

[1]In 2019 USD.

All estimates are rounded to the nearest 1,000.

The total price elasticities of demand for cigarettes for individuals in the poorest two quintiles are 2/3rd the value of the elasticities in the main analysis. These adjustments are expected to capture potentially higher levels of addiction to tobacco among the poor.

Next, we present results for women. Data from the 2016 DHS indicate that about 1% of women in Ethiopia smoked cigarettes (CSA and ICF, 2016). Table A6 below presents parameters for women in different age-wealth groupings. Unfortunately, the DHS did not collect smoking data from women in as much detail as it did from men. For female smokers, we had cigarette consumption levels for everyday smokers, but no information for occasional smokers. For this latter group, we assumed that their daily consumption was half the median number of cigarettes consumed per day by the regular female smokers (for men in the main analysis, typical cigarette consumption in each age-wealth category was the average consumption across regular and occasional smokers). Also, since the DHS interviewed women only up to the age of 49 years, we did not have smoking data for older women (in contrast, the DHS interviewed men up to age 59 years). Therefore, we excluded the older age groups from the studied female sample.

Table A6: Detailed input parameters used in model by age and wealth - women

Panel A: Female population distribution (proportions)

	Poorest	Poorer	Middle	Richer	Richest
0-4	0.017	0.016	0.015	0.013	0.011
5-9	0.019	0.018	0.017	0.016	0.010
10-14	0.015	0.015	0.015	0.016	0.013
15-19	0.007	0.008	0.009	0.010	0.014
20-24	0.007	0.007	0.007	0.008	0.011
25-29	0.007	0.008	0.008	0.007	0.012
30-34	0.006	0.006	0.006	0.006	0.008
35-39	0.005	0.004	0.006	0.006	0.007
40-44	0.003	0.003	0.003	0.004	0.004
45-49	0.002	0.003	0.003	0.003	0.003

Panel B: Female smoking prevalence (proportions)

	Poorest	Poorer	Middle	Richer	Richest
15-19	0.000	0.000	0.000	0.000	0.000
20-24	0.006	0.011	0.032	0.016	0.006
25-29	0.010	0.011	0.018	0.016	0.001
30-34	0.014	0.007	0.026	0.000	0.007
35-39	0.008	0.022	0.000	0.010	0.005
40-44	0.004	0.000	0.000	0.007	0.000
45-49	0.000	0.015	0.015	0.033	0.026

Panel C: Average per capita daily cigarette consumption

	Poorest	Poorer	Middle	Richer	Richest
15-19	0	0	0	0	0
20-24	2	2	2	2	4
25-29	2	2	2	2	5
30-34	1	2	3	0	3
35-39	2	2	2	2	1
40-44	3	2	2	2	3
45-49	9	2	3	2	2

Source: The 2016 Ethiopian Demographic and Health Survey (DHS). Sample weights used for all statistics derived from the DHS.
Note: the DHS did not survey girls below the age of 15 years and so we did not have data on smoking prevalence and total cigarette consumption for these groups. We assumed that children and youth aged below 15 years are yet to initiate smoking but that in the absence of the tax increase, their future tobacco use patterns would have been akin to those currently observed amongst individuals aged 15-24 years.

Below are results obtained for the female population. Estimates are lower for women relative to men largely due to significantly lower smoking prevalence in the former. Importantly, groups that tend to be most price elastic – i.e. the youth and the poor – are much less likely to be smokers in the female population. Also, note that we estimated potential effects for a smaller age group of women, those aged up to 49 years (due to lack of data for older women). Middle-income women demonstrated the highest smoking prevalence (Table A6) and were therefore likely to incur the greatest share of the potential health gains, increases in tax revenue contributions, cigarette expenditure savings and catastrophic health expenditures benefits.

Table A7: Projected effects of Ethiopia's tobacco tax increase, disaggregated by wealth quintile - Women

	Wealth quintile					
Outcome	Total	Poorest	Poorer	Middle	Richer	Richest
Years of life gained	1,118,000	165,000	223,000	454,000	208,000	68,000
Change in annual tax revenues[1]						
In year 1	893,000	30,000	97,000	268,000	250,000	248,000
Change in annual expenditures on cigarettes[1]						
In year 1	-1,027,000	-236,000	-283,000	-408,000	-108,000	7,000
Catastrophic health expenditures averted						
At 10% threshold	23,000	4,000	5,000	10,000	3,000	1000
At 25% threshold	10,000	2,000	2,000	4,000	2,000	0
At 40% threshold	8,000	1,000	2,000	4,000	1000	0

[1]In 2019 USD.
All estimates are rounded to the nearest 1,000.

Next, we looked at the changes in tobacco tax revenues and cigarette expenditures following the tax reform, and approximated the likely split due to former vs. continuing smokers. For example, we estimate the extent to which the tax burden of continuing smokers changes in the aftermath of the tax hike.

We begin by calculating the drops in revenues that former smokers in each group would be responsible for ($\Delta Q\_revenue(a,i)$). This is what these individuals were paying prior to the tax reform, but which they now would no longer pay. $\Delta C\_revenue\,(a,i)$ is the change in revenues borne by continuing smokers in the aftermath of the tax reform.

$$\Delta Q\_revenue(a,i) = Q(a,i) \times C_1(a,i) \times t_1 \quad . \qquad (14)$$

$$\Delta C\_revenue\,(a,i) = \Delta Tax\_revenue + \Delta Q\_revenue(a,i) \quad . \qquad (15)$$

Calculations for the split in cigarette expenditure changes are conducted analogously. Results are provided below in Table A8.

Table A8: Projected effects of Ethiopia's cigarette tax increase, disaggregated by wealth quintile - Split across quitters and continuing smokers

	Wealth quintile					
Outcome	Total	Poorest	Poorer	Middle	Richer	Richest
Change in annual tax revenues in year 1						
Total	26,173,000	2,218,000	3,432,000	4,545,000	2,361,000	13,617,000
Change for quitters	-4,758,000	-1,366,000	-1,227,000	-797,000	-285,000	-1,082,000
Change for continuing smokers	30,931,000	3,585,000	4,659,000	5,342,000	2,646,000	14,699,000
Change in annual expenditures on cigarettes in year 1						
Total	-18,544,000	-10,483,000	-8,013,000	-2,952,000	-338,000	3,242,000
Change for quitters	-26,648,000	-7,652,000	-6,872,000	-4,465,000	-1,596,000	-6,062,000
Change for continuing smokers	8,103,000	-2,831,000	-1,141,000	1,513,000	1,258,000	9,304,000

All values are presented in 2019 USD.

All estimates are rounded to the nearest 1,000.

References

Assebe, L. F., Negussie, E. K., Jbaily, A., Tolla, M. T. T., & Johansson, K. A. (2020). Financial burden of HIV and TB among patients in Ethiopia: a cross-sectional survey. *BMJ Open*, *10*(6), e036892.

Becker, G. S., Grossman, M., & Murphy, K. M. (1990). *An empirical analysis of cigarette addiction* (No. w3322). National Bureau of Economic Research.

Berg, G. D., & Kaempfer, W. H. (2001). Cigarette demand and tax policy for race groups in South Africa. *Applied Economics*, *33*(9), 1167-1173.

Central Statistical Agency – Ethiopia (CSA) and ICF. (2016). Ethiopia Demographic and Health Survey 2016. Addis Ababa, Ethiopia, and Rockville, Maryland, USA: CSA and ICF.

Chaloupka, F. J., & Wechsler, H. (1997). Price, tobacco control policies and smoking among young adults. *Journal of Health Economics*, *16*(3), 359-373.

Chaloupka, F. J., Yurekli, A., & Fong, G. T. (2012). Tobacco taxes as a tobacco control strategy. *Tobacco control*, *21*(2), 172-180.

Chelwa, G., & van Walbeek, C. (2019). Does cigarette demand respond to price increases in Uganda? Price elasticity estimates using the Uganda National Panel Survey and Deaton's method. *BMJ Open*, *9*(3), e026150.

Dauchy, E., & Ross, H. (2022). Is Illicit Cigarette Market a Threat to Tobacco Control in Ethiopia?. *Nicotine & Tobacco Research*, Forthcoming.

Doll, R., Peto, R., Boreham, J., & Sutherland, I. (2004). Mortality in relation to smoking: 50 years' observations on male British doctors. *BMJ*, *328*(7455), 1519.

Economics of Tobacco Control in South Africa Project (1998). The economics of tobacco control in South Africa. Report submitted to the International tobacco Initiative. Cape Town, Applied Fiscal Research Centre, School of Economics, University of Cape Town.

Emery, S., Ake, C. F., Navarro, A. M., & Kaplan, R. M. (2001). Simulated effect of tobacco tax variation on Latino health in California. *American journal of preventive medicine*, *21*(4), 278-283.

Erku, D. A., & Tesfaye, E. T. (2019). Tobacco control and prevention efforts in Ethiopia pre-and post-ratification of WHO FCTC: Current challenges and future directions. *Tobacco Induced Diseases*, *17*.

Ethiopia NCDI Commission. (2018). The Ethiopia Noncommunicable Diseases and Injuries (NCDI) Commission Report Summary: Addressing the Impact of Noncommunicable Diseases and Injuries in Ethiopia. Available from: http://www.ncdipoverty.org/ethiopia-report/.

Federal Democratic Republic of Ethiopia. (2016). Household Consumption Expenditure (HCE) Survey. Addis Ababa: Federal Democratic Republic of Ethiopia Central Statistics Agency.

Federal Democratic Republic of Ethiopia. (2019). Food and Medicine Administration Proclamation No. 1112/2019. Available from: https://www.tobaccocontrollaws.org/files/live/Ethiopia/Ethiopia%20-%202019%20Proclamation%20-%20national.pdf.

Fekadu, L., Hanson, C., Osberg, M., Makayova, J., Mingkwan, P., & Chin, D. (2017). Increasing access to tuberculosis services in Ethiopia: findings from a patient-pathway analysis. *The Journal of infectious diseases*, *216*(suppl_7), S696-S701.

Gandini, S., Botteri, E., Iodice, S., Boniol, M., Lowenfels, A. B., Maisonneuve, P., & Boyle, P. (2008). Tobacco smoking and cancer: a meta-analysis. *International journal of cancer*, *122*(1), 155-164.

Hailu, A., & Mariam, D. H. (2013). Patient side cost and its predictors for cervical cancer in Ethiopia: a cross sectional hospital-based study. *BMC Cancer*, *13*(1), 69.

Ho, L. M., Schafferer, C., Lee, J. M., Yeh, C. Y., & Hsieh, C. J. (2017). The effect of cigarette price increases on cigarette consumption, tax revenue, and smoking-related death in Africa from 1999 to 2013. *International Journal of Public Health*, *62*(8), 899-909.

Institute for Health Metrics and Evaluation (IHME). (2019). GBD Compare Data Visualization. Seattle, WA: IHME, University of Washington. Available from http:// vizhub.healthdata.org/gbd-compare. Accessed December 2, 2020.

International Agency for Research on Cancer (IARC). (2011). *Effectiveness of tax and price policies for tobacco control,* Vol. 14. World Health Organization.

James, E. K., Saxena, A., Restrepo, C. F., Llorente, B., Vecino-Ortiz, A. I., Uribe, M. V., ... & Verguet, S. (2019). Distributional health and financial benefits of increased tobacco taxes in Colombia: results from a modelling study. *Tobacco control*, *28*(4), 374-380.

Jha, P., Joseph, R., Li, D., Gauvreau, C., Anderson, I., Moster, P., & Bonu, S. (2012). *Tobacco Taxes: A Win-win Measure for Fiscal Space and Health. November 2012*. Asian Development Bank.

Joossens, L., Lugo, A., La Vecchia, C., Gilmore, A. B., Clancy, L., & Gallus, S. (2014). Illicit cigarettes and hand-rolled tobacco in 18 European countries: a cross-sectional survey. *Tobacco control*, *23*(e1), e17-e23.

Kidane, A., Mduma, J., Naho, A., Ngeh, E. T., & Hu, T. W. (2015). The demand for cigarettes in Tanzania and implications for tobacco taxation policy. *Advances in Economics and Business*, *3*(10), 428.

Kiros, M., Dessie, E., Jbaily, A., Tolla, M. T., Johansson, K. A., Norheim, O. F., ... & Verguet, S. (2020). The burden of household out-of-pocket health expenditures in Ethiopia: estimates from a nationally representative survey (2015–16). *Health Policy and Planning*, *35*(8), 1003-1010.

Laxminarayan, R., & Deolalikar, A. (2004). Tobacco initiation, cessation, and change: evidence from Vietnam. *Health economics*, *13*(12), 1191-1201.

Marquez, P.V. (2019). Ethiopia's New Tobacco Control Law: A Step Forward that Needs to be Complemented by Higher taxes. The World Bank. Available from: https://blogs.worldbank.org/health/ethiopia-s-new-tobacco-control-law-step-forward-needs-be-complemented-higher-taxes.

Mukong, A. K., & Tingum, E. N. (2020). The demand for cigarettes: New evidence from South Africa. *Development Southern Africa*, *37*(1), 40-54.

Postolovska, I., Lavado, R., Tarr, G., & Verguet, S. (2018). The health gains, financial risk protection benefits, and distributional impact of increased tobacco taxes in Armenia. *Health Systems & Reform*, *4*(1), 30-41.

Reekie, W. D. (1994). Consumers' surplus and the demand for cigarettes. *Managerial and Decision Economics*, *15*(3), 223-234.

Shimkhada, R., & Peabody, J. W. (2003). Tobacco control in India. *Bulletin of the World Health Organization*, *81*, 48-52.

Stoklosa, M. (2020). Ethiopia is Increasing its Tobacco Taxes by Introducing a Specific Excise. The Tobacco Atlas. Available from: https://tobaccoatlas.org/2020/02/25/ethiopia-is-increasing-its-tobacco-taxes-and-price-by-introducing-a-specific-excise/.

Tolla, M. T., Norheim, O. F., Verguet, S., Bekele, A., Amenu, K., Abdisa, S. G., & Johansson, K. A. (2017). Out-of-pocket expenditures for prevention and treatment of cardiovascular disease in general and specialised cardiac hospitals in Addis Ababa, Ethiopia: a cross-sectional cohort study. *BMJ Global Health*, *2*(2).

United Nations. (2019). World population prospects 2019. Available from https://population.un.org/wpp/.

van Walbeek, C. P. (1996). Excise taxes on tobacco: how much scope does the government have?. *South African Journal of Economics*, *64*(1), 20-42.

Verguet, S., Gauvreau, C. L., Mishra, S., MacLennan, M., Murphy, S. M., Brouwer, E. D., ... & Jamison, D. T. (2015). The consequences of tobacco tax on household health and finances in rich and poor smokers in China: an extended cost-effectiveness analysis. *The Lancet Global Health*, *4*(3), e206-e216.

Watkins, D. A., Qi, J., Kawakatsu, Y., Pickersgill, S. J., Horton, S. E., & Jamison, D. T. (2020). Resource requirements for essential universal health coverage: a modelling study based on findings from Disease Control Priorities. *The Lancet Global Health*, *8*(6), e829-e839.

World Bank. (2020). World Development Indicators. Available from https://datacatalog.worldbank.org/dataset/world-development-indicators.

Young, D., Yong, H. H., Borland, R., Ross, H., Sirirassamee, B., Kin, F., ... & O'Connor, R. (2008). Prevalence and correlates of roll-your-own smoking in Thailand and Malaysia: Findings of the ITC-South East Asia Survey. *Nicotine & tobacco research*, *10*(5), 907-915.
